# Lymphatic Endothelial Cells and Organ-Associated Lymphangiogenesis in Tumor Microenvironment

**DOI:** 10.3390/cells15010028

**Published:** 2025-12-23

**Authors:** Rui-Cheng Ji

**Affiliations:** Faculty of Welfare and Health Science, Oita University, Oita 870-1192, Japan; ji@oita-u.ac.jp; Tel./Fax: +81-097-554-7973

**Keywords:** lymphangiogenesis, lymphatic endothelial cells, cancer metastasis, tumor microenvironment, immunotherapy

## Abstract

**Highlights:**

**What are the main findings?**

- LECs have heterogeneous and plastic features.
- Lymphatic vessels have a dual role in cancer progression and metastasis.
- Organ-associated lymphangiogenesis is regulated by various signaling pathways in TME.

**What are the implications of the main findings?**

- Functional diversity of LECs will contribute to elucidating the pathogenic mechanism of lymphatic-related diseases.
- Lymphatic modulation is focused on inhibiting pro-metastatic functions and enhancing protective anti-tumor immunity.
- Intervention of organ-specific lymphangiogenesis may be promising for molecular target and gene therapy.

**Abstract:**

Lymphatic vessels are a kind of heterogeneous and versatile component of the lymphatic system, with a unique ability to respond to environmental changes in different organs. The heterogeneity and plasticity of lymphatic endothelial cells (LECs) and defective lymphatic architecture are critical for organ-specific lymphatic function. Moreover, lymphatic vessels have a dual effect on tumor microenvironment (TME), and lymphangiogenesis, an active and dynamic player, is a hallmark of cancer progression and treatment resistance. Dysregulation of lymphatic vessels and uncontrolled lymphangiogenesis contribute to the pathogenesis of many diseases, including cancer. Increasing evidence has indicated that lymphangiogenesis provides a critical target for inhibiting lymphatic metastasis, in which immune checkpoint inhibitors, either alone or combined with chemotherapy, may have a therapeutic value. This article reviews the current status of tumor-associated lymphangiogenesis and lymphatic remodeling, as well as the crosstalk among LECs, immune cells and cancer cells, which will help to further understand the role of lymphangiogenesis in cancer progression, metastasis, and therapy.

## 1. Introduction

**Figure 1:**
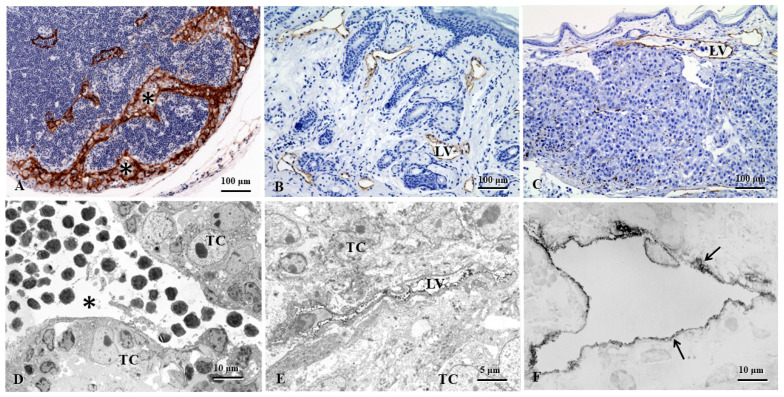
The lymphatic morphological changes occur in some pathological conditions, e.g., melanoma, hybridoma, and lymphangiomas. (**A**). The mouse LN shows greatly expanded subcapsular and cortical sinuses (asterisks) filled with or adjacent to accumulated melanoma cells. (**B**). In a complete Freund’s adjuvant (CFA)-induced model of non-obese diabetic (NOD) mice, obviously increased lymphatic vessels, so-called lymphangiomas, are found in the dermal and subcutaneous tissues. (**C**). In the melanoma mouse model, dermal lymphatic vessels are found in transporting tumor cells. (**D**). The popliteal LN of the melanoma mouse model shows intranodal lymphatic sinuses surrounded by metastasized tumor cells. (**E**). In the subcutaneous tissues of the melanoma mouse model, the intratumoral lymphatic vessel is collapsed due to surrounding metastasized tumor cells. (**F**). In the intestine of hybridoma mouse model, the transmission electron microscopy imaging shows the LECs (arrows) with pre-embedding immunostaining. (**A**–**C**,**F**): LYVE-1 immunohistochemical staining; (**D**,**E**): 5′-nucleotidase (5′-Nase)-cerium histochemical staining. LV: lymphatic vessel; TC, Tumor cells [1,2].

Lymphatic vessels transport extravasated tissue fluid, macromolecules, and immune cells from peripheral tissues to lymph nodes (LNs) and, finally, back to the systemic circulation. The lymphatic morphological changes occur in some pathological conditions, e.g., melanoma, hybridoma, and lymphangiomas (Figure 1) [1,2]. Lymphatic vessels actively participate in the regulation of tumor cell behaviors and modulation of antitumor immune responses. Understanding functional heterogeneity and plasticity of tumor lymphangiogenesis will help to explore the underlying molecular mechanisms of cancer progression and metastasis. Lymphangiogenesis in tumor-draining LNs occurs even before cancer cells arrive at the regional or sentinel LNs. LN lymphangiogenesis may further act as a permissive lymphovascular niche for metastatic cell survival [3]. In the tumor microenvironment (TME), the interaction of LECs with immune cells and tumor cells greatly impacts lymphatic function. Thus, the lymphatic vessel is emerging as a multifaceted regulator of tissue fluid homeostasis, modulation of immune response, and tumor growth and tumor cell dissemination.

In recent years, single-cell RNA sequencing (ScRNA-seq) technology has become the state-of-the-art approach for unraveling the heterogeneity of organ-specific endothelial cells (ECs) in health and disease. The heterogeneity of LECs is closely associated with their functional diversities and interacts with immune cells in TME. In LNs, LEC subsets exhibit differences in gene expression relating to specific functions and locality, facilitating innate and acquired immune responses through antigen presentation, and LN remodeling and regulation of leukocyte entry and exit [4]. Modulating lymphatic function to reduce metastatic spread has shown great therapeutic potential in targeting tumor lymphangiogenesis. Organ-associated lymphangiogenesis displays heterogeneity in cellular origins and functional plasticity in tumor tissues. This review has summarized the research advances of functional complexity and multiplicity of LECs in cancer progression. The mechanism that causes lymphangiogenesis heterogeneity in solid tumors has been elucidated in the context of providing better insight into potential implications for cancer treatment.

## 2. The Heterogeneity and Functional Plasticity of Tumor-Associated Lymphatic Vessels

The lymphatic vessel is crucial for maintaining tissue fluid homeostasis, providing immune surveillance, and mediating lipid absorption. The lymphatic system exhibits distinct morphological and functional features with LEC adaptation to various pathological events.

### 2.1. Endothelial Cells (ECs)

EC heterogeneity has been observed across developmental, physiological, and pathological conditions. ECs can adopt subtly different phenotypes in response to the changing extracellular environment. Accumulating evidence suggests that differentiated ECs exhibit remarkable heterogeneity and plasticity. In the adult, maintenance of a differentiated endothelial state is an active process requiring constant signaling input. The failure to do so leads to the development of endothelial-to-mesenchymal transition (EndMT), a dynamic process contributing to the pathogenesis of several diseases. During this process, the endothelium undergoing EndMT presents dismantled junctions and cytoskeletal contractility, favoring the passage of tumor cells [5]. A transient mesenchymal activation concomitant with a metabolic adaptation may facilitate endothelial cell migration and clonal expansion to regenerate the vascular network. EndMT not only participates in embryonic development processes, but also in the pathogenesis of genetically determined and acquired diseases, e.g., inflammation, fibrotic disorder, and cancer [6,7,8]. Thus, targeted manipulation of cell fate may intercross with lymphangiogenesis-associated pathological conditions, e.g., inflammation, tumorigenesis, and wound healing [9].

EC phenotypes reflect varying responses to different stimuli and changes in the pathological environment. The local microenvironment elicits heterogeneous endothelial cell phenotypes determined by local needs. This heterogeneity also explains the diverse pathological responses to a disturbed endothelial integrity [10]. The occurrence of blood endothelial cell (BEC)/LEC lineage switches in malignancies has indicated that an abnormal environment can trigger the dedifferentiation of LECs into BECs by altering the normal levels of prospero homeobox 1 (Prox-1) expression. In angiosarcomas, intratumoral capillaries express a mixed blood/lymphatic endothelial phenotype. These ECs may undergo reprogramming and form aberrant phenotypes co-expressing specific markers for BECs and LECs [11,12,13]. Lymphangiogenic molecules are supposed to be involved in the pathogenesis of Kaposi sarcoma, in which the sarcoma herpesvirus induces transcriptional drift in both LECs and BECs [14]. In the study of endothelial cells, a lipid raft-targeted therapeutic approach is suggested due to the induction of low-density lipoprotein receptor-related protein 8 (LRP8) by anti-β2 glycoprotein I (anti-β2-GPI) antibodies [15]. However, the involvement of lipid-raft-dependent signaling in LEC biology or tumor-associated lymphangiogenesis still remains to be explored.

Podoplanin-expressing cancer-associated fibroblasts (CAFs) are associated with cancer cell proliferation, invasion, and metastasis in several types of tumors. In human intrahepatic cholangiocarcinoma (CCA), podoplanin-positive CAFs may transform into LECs through mesenchymal-to-endothelial transition (MEndT), contributing to lymphangiogenesis [16]. Vice versa, in the melanoma model, the transforming growth factor-β1 (TGF-β1) induces proliferating ECs to undergo a phenotypic conversion into fibroblast-like cells. EndMT is associated with the emergence of mesenchymal marker fibroblast-specific protein-1 (FSP1) and down-regulation of CD31 [6]. Collagen and calcium-binding EGF domain-1 (CCBE1) derived from CAFs and cancer cells promotes tumor lymphangiogenesis and lymphatic metastasis via enhancing vascular endothelial growth factor-C (VEGF-C) proteolysis and maturation, but it is negatively regulated by TGF-β signaling in colorectal cancer [17]. Recently, hypoxic CAFs have been revealed to enhance colorectal cancer lymphatic metastasis via C-type lectin domain containing 11A/leucine-rich repeat-containing G-protein-coupled receptor 5 (CLEC11A/LGR5)-mediated Wingless (WNT)/β-catenin signaling pathway that promotes epithelial–mesenchymal transition (EMT) and lymphangiogenesis [18]. In lung adenocarcinoma, CAF-derived extracellular superoxide dismutase (SOD3) enhances EMT and lymphangiogenesis to drive lymph node metastasis [19]. CAFs can be reprogrammed by cancer cells, leading to the production of extracellular vesicles (EVs) that serve as carriers for bioactive substances, including proteins, nucleic acids, and metabolic products. Therefore, by delivering specific miRNAs, CAF-derived EVs can modulate lymphangiogenesis and tumor progression [20]. Heterogeneous EVs from various types of tumors are crucial for inducing the formation of pre-metastatic niches in draining LNs. Integrin α6-containing EVs promote lymphatic remodeling for pre-metastatic niche formation in LNs via interplay with CD151 [21]. Secreted EVs promote distal organ metastasis and influence TME, where endothelial cells exhibit distinct functional and molecular heterogeneity depending on vessel and organ types, and individual age. Understanding EC heterogeneity may facilitate optimal selection of therapeutic approaches and improve outcomes of various organ-specific diseases.

### 2.2. ScRNA-Seq of LECs

LECs are involved in immune cell migration, antigen presentation, and cancer cell metastasis. LECs show different, unique properties due to their organ-specific identity and environmental preferences. In recent years, the development of ScRNA-seq techniques has greatly increased our knowledge of cellular heterogeneity, cell subtypes, and states by tissue decomposition, especially on new functions of LECs in health and disease [22,23,24]. LECs are functionally and anatomically distinct from BECs. ScRNA-seq of ECs isolated from adult male mice has identified transcriptomic signatures of quiescent arterial, capillary, venous, and LECs in different tissues. ECs from different tissues exhibit prominent transcriptomic heterogeneity [25]. Moreover, single-cell transcriptomic profiling unveils molecular features of LECs in different tissues, contributing to tissue-specific specialization, e.g., skin, intestines, and meninges. Inflammation induces basement membrane degradation of dermal collecting lymphatic vessels and preferential upregulation of dendritic cell (DC) trafficking molecule, the vascular cell adhesion molecule-1 (VCAM-1) [26]. Folliculin, a tumor suppressor protein, maintains the separation of blood vessels and lymphatic vessels by limiting the plasticity of committed ECs [27]. In tumors, ScRNA-seq analysis shows a heterogeneous gene expression signature of ECs, leading to the identification of metabolic plasticity and collagen modification as potential critical angiogenic players [28].

Single-cell profiling of LECs also unravels the molecular heterogeneity and niche-specific specialization of lymphatic sinuses in LNs. ScRNA-seq of human LNs unveils six types of LECs located in different sites with distinct molecular signatures. LECs lining the floor and ceiling of the subcapsular sinus, medullary sinus, and valve are the main types expressing different chemokines, in which LECs of the subcapsular sinus floor and medullary sinus are specialized to regulate immune responses [29]. Deletion of autophagy-related gene 5 (ATG5) causes a dominant downregulation of several genes relating to ceiling LEC proliferation, suggesting a pro-lymphangiogenic action of ATG5 in ceiling LECs. Tumor-draining LNs further show that loss of ATG5 remodels niche-specific LEC phenotypes involved in molecular pathways regulating lymphocyte trafficking and LEC-T cell interactions [30]. Therefore, LECs in LNs show multiple cell subsets and a unique set of genes depending on intranodal residence and location. Single-cell transcriptomics of LECs has indicated that the cell subsets as modulation targets are involved in various physiological and pathological functions in different tissues.

Circular RNAs (circ-RNAs) involved in the regulation of tumor-associated lymphangiogenesis and LN metastasis have shown different dependence on growth factors in the cancers of the pancreas, ovary, and urinary bladder. Circ-NFIB1 reduces the oncogenic effect of miR-486-5p, but upregulates PIK3R1 expression, which downregulates VEGF-C expression through inhibition of the PI3K/Akt pathway and then suppresses lymphangiogenesis and LN metastasis in pancreatic ductal adenocarcinoma [31]. In ovarian cancer, downregulated circular RNA absent-small-homeotic-2-like protein (circ-ASH2L) is shown to inhibit lymphangiogenesis, and its upregulation plays an oncogenic role via competition with VEGF-A for binding to miR-665 [32]. In bladder cancer, circ-EHBP1 promotes lymphangiogenesis and LN metastasis via miR-130a-3p/TGF-βR1/VEGF-D axis, but independent of VEGF-C [33].

### 2.3. Lymphatic Heterogeneity and Plasticity

Organ-associated lymphatic vessels show endothelial heterogeneity and plasticity in different physiological and pathological processes, reflecting their functional specialization to control the tissue microenvironment. The heterogeneity of endothelial junctions is especially reflected by differential expression and specific subcellular localization. Junctional adhesion molecule-2 (JAM-2), a member of the immunoglobulin superfamily, is expressed on LECs and high endothelial venules in LNs, indicating that the intercellular junctions possess a highly permeable feature [34]. LECs require constant expression of a certain gene to maintain their phenotypic identity. During the process, the lymphatic-specific homeobox transcription factor Prox-1 is absolutely required; conditional down-regulation of Prox-1 in embryonic, postnatal, or adult mice results in a reversion of LECs back to BECs [35]. Thus, alternative developmental origins of LECs in some organs may contribute to the diversity of their functions in adult tissues [36]. Although VEGFR-3 deletion results in lymphatic hypoplasia in mouse embryos, incomplete deletion or mosaic loss of VEGFR-3 during postnatal development may lead to excessive lymphangiogenesis. In this case, the heterogeneity in VEGFR-3 levels drives lymphatic hyperplasia through cell-autonomous and non-cell-autonomous mechanisms during pathological tissue growth [37]. Forkhead box protein C2 (Foxc2) controls LEC specialization and function in adulthood. Foxc2 loss disrupts the specialization of LEC subsets, leading to the emergence of mixed or pro-fibrotic cell populations. Foxc2 inactivation also disrupts the hierarchical organization of the lymphatic network in an organ-specific manner [38]. Additionally, embryonic dermal lymphatic vessels express NOTCH4 and the Notch ligand, DLL4. Unexpectedly, Notch4 signaling and canonical Notch signaling have distinct functions in the coordination of embryonic dermal lymphangiogenesis [39]. The heterogeneity of lymphatic vessels with organ-specific structural and functional features may be due to the diverse developmental origins and molecular specializations of LECs to adapt to local environmental requirements during physiological and pathological processes [36,40].

Lymphangiogenesis shows heterogeneity and functional plasticity, usually in structures, gene expression profiles, and cellular origins. The plasticity of differentiated LECs may help explain the role of lymphangiogenesis in tumor metastasis. Tumor-associated lymphangiogenesis, although sharing some common biological markers and regulatory mechanisms, displays different transcriptional and molecular profiles. In myocardial wound repair, the lymphangiogenic response occurs from the podoplanin-positive cell population, showing highly heterogeneous and endothelial commitment [41]. In the central nervous system (CNS) of adult mice, VEGF-C/VEGFR-3 signaling regulates meningeal lymphangiogenic responses and lymphatic regression, and thus the lymphatic plasticity allows involvement of cerebrospinal fluid (CSF) drainage and neuropathological processes [42]. Long-term exercise protects against Alzheimer’s disease by enhancing the plasticity and drainage of meningeal lymphatic vessels through downregulation of the eleven-nineteen lysine-rich leukemia-associated factor-2 (EAF-2)-p53-thrombospondin-1 (TSP-1) pathway associated with reactive astrocytes [43]. Age-related meningeal extracellular matrix (ECM) remodeling can compromise the lymphatic function of the CNS. In the mouse model with constitutively active TGF-β receptor 1 (TGFβR1) signaling in dural fibroblasts, ECM stiffness can disrupt endothelial junction formation and reduce lymphangiogenesis. Thus, the excessive peri-lymphatic collagen deposition caused by fibroblast-mediated matrix remodeling impairs meningeal lymphatic drainage and CSF clearance, and alters meningeal immunity [44]. Although LECs from different organs display considerable variation, it is still unclear to what extent functional properties of organ-specific endothelial cells are intrinsic, acquired, and/or reprogrammable [45].

### 2.4. Tumor Microenvironment (TME) and Lymphatic Remodeling

TME is a dynamic ecosystem that includes a diversity of immune cells, CAFs and ECs, and soluble factors, signaling molecules, as well as ECM. Tumor-specific T and B cell immunity may induce some of the molecular factors required for the formation and maintenance of tertiary lymphoid structure (TLS) [46]. TLS with heterogeneity of B cell subtypes facilitates immune cell recruitment to TME and affects the outcome of muscle-invasive bladder cancer [47]. CAFs, an essential component of TME, show heterogeneity and plasticity across different cancer types, and greatly influence cancer hallmarks [48].

Lymphangiogenesis and lymphatic remodeling are involved in tumor progression and metastasis, and immunotherapeutic outcomes. Disorganized lymphatic vessels regulate tumor immune response and constitute an important factor for modulating TME. Dynamic interactions between tumor cells and LECs have greatly affected malignant evolution. WNT5B, a key molecular factor in TME, involves bidirectional crosstalk between melanoma cells and LECs and promotes melanoma metastasis. The molecular and functional changes in LECs induced by melanoma cells are mediated by the Delta-like protein 4 (DLL4)/Notch3/WNT5B signaling axis [49]. In orthotopic tumor models, LECs restrain tumor immunity via programmed cell death-ligand 1 (PD-L1), inducing apoptosis in tumor-specific CD8^+^ central memory cells in tumor-draining LNs [50]. Anexelekto (AXL), a tyrosine kinase receptor, promotes lymphangiogenesis by amplifying the VEGF-C-mediated protein kinase B (AKT) pathway. AXL-expressing LECs are infiltrated into LN parenchyma with increased density and penetration extent under metastatic conditions, suggesting an intricate interplay between AXL signaling and lymphatic changes in structure and function in TME [51]. During VEGF-D-driven metastasis, LECs from collecting lymphatic vessels have already altered their gene signature [52], reflecting that lymphatic subtypes in TME may respond to different spread patterns. In an orthotopic mouse model of breast cancer, VEGF-C derived from tumor cells is required for chronic stress to induce increased intratumoral lymphatic vessel density (LVD) and lymphatic dilation, depending on cyclooxygenase-2 inflammatory signaling from tumor-associated macrophages (TAMs) [53]. In TME, abnormal proliferation or alteration of initial lymphatic vessels enables tumor cells to access lymphatic drainage, and dilation of collecting lymphatic vessels within tumor tissues or beyond further facilitates tumor cell trafficking to the sentinel LNs. LECs in tumor tissues and draining LNs undergo substantial remodeling in response to oncogenic signals from TME. Definitely, LECs have heterogeneous and plastic features in health and disease, and their functional diversity in response to different stimuli and changes in the microenvironment will contribute to elucidating the pathogenic mechanism of lymphatic-related diseases.

## 3. Tumor Lymphangiogenesis and Immunotherapy

Functional lymphatic vessels are essential for the initiation of anti-tumor immunity responses, and cancer immunotherapy delivery mediated by cytokine, chemokine, and adhesion molecule expression. Lack of functional intratumoral lymphatic vessels inhibits the clearance of extracellular fluid, further contributing to high interstitial pressure within tumors [54]. LECs upregulate immunoregulatory molecules, e.g., PD-L1 and major histocompatibility complex class II (MHC-II), to induce T cell-mediated immunosuppression in tumor tissues. At different cascade levels, the specific targeting of lymphangiogenesis via inhibiting VEGF-C/VEGFR-3 signaling pathway is fundamentally depending on the profile of organ-associated LEC specialization and heterogeneity for preventing tumor progression and metastasis. In recent years, the expression of microRNAs in LECs has been indicated to promote or inhibit lymphangiogenesis in different types of cancers. Thus, a better understanding of the complex interplay between LECs and their contribution to TME is required to develop novel strategies for cancer immunotherapy.

### 3.1. Dual Role of Lymphatic Vessels in Cancer Progression and Metastasis

LECs play a dual role in promoting and hindering anti-tumor immune responses during tumor progression. Tumor-associated lymphangiogenesis can facilitate tumor cell dissemination and metastasis via immune evasion. The immunosuppressive signals from LECs can inhibit immune cell activation, impair effector function, and promote immune tolerance to the tumor antigen. Moreover, immunosuppressive factors delivered through lymphatic flow from tumor tissues can attenuate LEC-mediated anti-tumor immunotherapy [55,56]. The impact of tumor-associated lymphangiogenesis in immune evasion and immunosuppression can facilitate tumor metastasis, but lymphatic vessels in TME can support adaptive anti-tumoral immune responses by transporting immune cells and antigens from the tumor to the draining LNs [57]. Intradermally implanted melanoma in the K14-VEGFR3-Ig mouse model that lacks dermal lymphatic vessels exhibits rapid tumor cell growth but decreased distant metastasis, which results from impaired antitumor immunity characterized by reduced cytokine expression and immune cell infiltration [57].

Increased VEGF-C expression and LVD in TME of human melanoma show significant positive correlation with tumor-infiltrating CD8^+^ T cells and expression of immunosuppressive molecules, e.g., inducible nitric oxide synthase (iNOS), indoleamine 2,3-dioxygenase (IDO), and arginase-1 (ARG-1) [58]. Tumor-associated LECs promote tumor progression and metastasis, and regulate antitumor immune responses. Interferon-γ (IFN-γ) released by effector T cells enhances the expression of immunosuppressive markers by tumor-associated LECs. T cell-based immunotherapy at higher effector T cell densities within the tumor induces LEC apoptosis and decreases tumor LVD, and consequently impairs lymphatic flow and reduces LN metastasis [59]. IFN-γ can upregulate inhibitory molecules, including IDO-1 and nitric oxide synthase 2 (NOS2), which impair T cell proliferation and response by interfering with metabolic pathways. In this way, IFN-γ and NOS2 expressions are essential to the immunosuppressive function of LECs [60].

The cross-presentation of tumor antigens and PD-L1 expression by LECs may promote immune tolerance. In melanoma models, LECs are responsible for the maintenance of peripheral tolerance by upregulating PD-L1 to inhibit T cell activation. T-cell inhibitory molecule PD-L1 upregulation on LECs may be stimulated by IFN-γ released by stromal cells in TME [61,62]. In malignant skin tumors, disruption of IFN-γ-dependent crosstalk through lymphatic-specific loss of IFN-γR enhances T cell accumulation, leading to increased tumor control [62]. In human esophageal squamous cell carcinoma, LEC-mediated accumulation of CD177+ regulatory T cells (Tregs) with high expression of immune inhibitory cytokine, interleukin-35 (IL-35), is related to exhaustion of tumor-infiltrating CD8^+^ T cells, in creating an immunosuppressive microenvironment to favor tumor progression and resistance to immunotherapy [63]. In human colorectal cancer and mouse tumor models, Tregs are recruited to the peri-lymphatic region in tumor stroma by mature DCs enriched in immunoregulatory molecules (mregDCs) to establish a Treg-mregDC-lymphatic niche for sustaining Treg activation. The activated Tregs further restrain tumor antigen trafficking to draining LNs and thereby impede the initiation of anti-tumor adaptive immune responses [64]. Antigen-specific CD8^+^ T cell accumulation in tumors is a prerequisite for effective immune checkpoint blockade. Tumor-associated lymphatic vessels control CD8^+^ T cell exit from tumors via the chemokine CXCL12. Loss of lymphatic-specific CXCL12 or CXCR4 inhibition boosts T cell retention and enhances tumor control. An approach to limit T cell egress may increase the quantity and quality of intratumoral T cells and thereby response to immunotherapy [65]. In mouse melanoma models, lymphangiogenesis-inducing vaccines elicit potent tumor-specific T cell immunity and provide effective tumor control and long-term immunological memory [66].

TME has a significant impact on tumor growth, progression, metastasis, and immunotherapy efficacy. Lymphatic vessels are relevant for tumor spread and immune modulation during therapy. However, it should be noted that dysfunction of lymphatic vessels, as a result of their altered structures during cancer metastasis and treatment, may lead to direct clinical consequences like lymphedema.

### 3.2. Lymphangiogenesis and Immunotherapy

Lymphatic vessels transport antigen and immune cells from peripheral tissues to LNs for initiating adaptive immune responses. LECs via expression of numerous immune mediators and growth factors can influence the immune function [67]. Two distinct differentiation lineages of tumor-associated LECs are described to be responsible for antigen presentation and lymphangiogenesis [68]. Antigen presentation by tumor LECs promotes intratumoral Treg suppressive function. Tumor-associated LECs significantly contribute to the generation and modification of immunosuppressive TME. Tumor-associated lymphangiogenesis promotes Treg accumulation, but dampens CD8^+^ effector T-cell infiltration in tumors, suggesting that tumoral LECs support an immunosuppressive microenvironment by impacting Tregs [69]. LECs play an important role in the maintenance of the immunosuppressive TME, where hypoxia is an important inducer of tumor lymphangiogenesis [70].

TAMs are believed to contribute to tumor progression and are generally characterized as M2-like macrophages. Macrophage plasticity and diversity provide a platform to remodel immunosuppressive TME in tumorigenesis and immunotherapy, in which the important effect of TAMs on lymphangiogenesis is by upregulating VEGF-C [71,72]. IL-10 and TGF-β are important factors for creating an immunosuppressive microenvironment. IL-10 may contribute to tumor immunosuppression by promoting the polarization of M2 macrophages within TLS in TME [73]. Blockade of TGF-β-mediated signaling pathways in TME can attenuate the immunosuppressive effects of adaptive or induced Tregs, resulting in increased antitumor immunity [74]. Colorectal cancer-derived exosomes can modify TME in adjacent organs and promote lymphangiogenesis in the sentinel LNs, facilitating distant metastasis via interferon regulatory factor 2 (IRF2) that further induces VEGF-C secretion by TAMs. IRF-2 knockdown attenuates lymphatic remodeling in the sentinel LNs and suppresses cancer metastasis [75]. Exosomes, small EVs, can mediate intercellular crosstalk within TME by transferring mRNAs, microRNAs, and proteins from donor to recipient cells. Cancer-secreted exosomal miR-1468-5p activates immunosuppressive reprogramming of lymphatic vessels via PD-L1 upregulation and lymphangiogenesis to impair CD8^+^ T cell immunity, and subsequently promotes tumor immune escape [76]. Tumor-associated LECs take on an immunosuppressive phenotype expressed by immune-inhibitory molecules, in which the programmed cell death 1 (PD-1) and its ligand PD-L1 are upregulated in tumor-associated lymphatic vessels [61,62]. PD-1/PD-L1 signaling regulates polarization of M2 TAMs. Inhibition of PD-L1 induces a decrease in M2 markers, IL-10, and Arg-1, but an increase in M1 markers, IL-12, and tumor necrosis factor-α (TNF-α) [77]. Immune checkpoint blockade therapy using antibodies to block receptor-ligand interactions, e.g., anti-PD-1 and anti-PD-L1, has gained ground and been approved for clinical use [78].

In melanoma, VEGF-C-mediated tumor lymphangiogenesis correlates with metastasis and poor prognosis, and promotes T-cell infiltration and potentiates immunotherapy. Increased T-cell activation via tumor antigen transport to draining LNs is associated with decreased metastatic burden. Lymphangiogenesis provides a critical target for therapeutic intervention to inhibit lymphatic metastasis. In human metastatic melanoma, serum VEGF-C concentration is associated with T cell activation and expansion after peptide vaccination, and clinical response to checkpoint blockade [79]. Lymphatic vessels can promote T cell recruitment to TME via CCL21/CCR7 signaling. VEGF-C, beyond inducing lymphangiogenesis, is associated with metastasis and poor prognosis, and promotes immune suppression, and interestingly, it can potentiate immunotherapy by attracting naïve T cells in melanoma [79]. Prophylactic VEGF-C treatment has been indicated to increase lymphangiogenesis in the meninges and, consequently, to evoke a robust and long-lasting T cell-dependent immune response against neoplasms. Therapeutic delivery of VEGF-C increases checkpoint inhibitor therapy to eradicate existing glioblastoma multiforme by increasing T cell priming and improving anti-tumor immunity, rather than inducing metastasis through lymphangiogenesis [80]. Anti-lymphangiogenesis seems violate the principle of immune checkpoint blockade. However, a recent study has indicated that anti-lymphangiogenesis therapy contributes to enhanced intratumoral retention of therapeutic drugs [81]. Moreover, 25-hydroxycholesterol produced by LECs in TME has a beneficial effect on mouse melanoma growth and response to immunotherapy, indicating extracellular oxysterols can prevent polarization of TAMs into immunosuppressive cells [82]. Finally, the lymphatic system acts as an autoimmune controller, playing a critical role in transporting self-antigens and maintaining peripheral tolerance. Recently, the therapeutic potential of targeting lymphatic proliferation by VEGFR-3 inhibition in LN has been indicated to attenuate autoimmune response, and even cancer-associated inflammatory processes [83]. Autoimmune pathologies, however, seem to be highly heterogeneous, showing a disease-specific up- and downregulation of VEGFR-3 signaling [84], which suggests that the choice of therapeutic approaches for promoting or inhibiting lymphangiogenesis should be made based on the type of disease. Clearly, the involvement of lymphangiogenesis in autoimmune disorders will also be an important and interesting research field. A successful immunotherapy is expected to effectively induce immune-mediated tumor cell death and regression by modulating processes of tumor initiation, promotion, and progression.

Lymphatic vessels act as a double-edged sword in cancer metastasis and therapy. Dynamic remodeling of lymphatic vessels and LN is greatly involved in cancer metastasis and immunotherapeutic outcomes, which can both promote metastasis and facilitate effective immunotherapy. Undoubtedly, intratumoral and peritumoral lymphatic vessels provide a direct route for cancer cells to escape the primary tumor and travel to regional LN, and even create a supportive environment for metastasis in LN. Current immunotherapy, therefore, focuses the strategy on inhibiting pro-metastatic functions of lymphatic vessels, while simultaneously enhancing their involvement in protective anti-tumor immunity.

## 4. Organ-Associated Lymphatic Vessels and Lymphangiogenesis in Cancers

**Figure 2:**
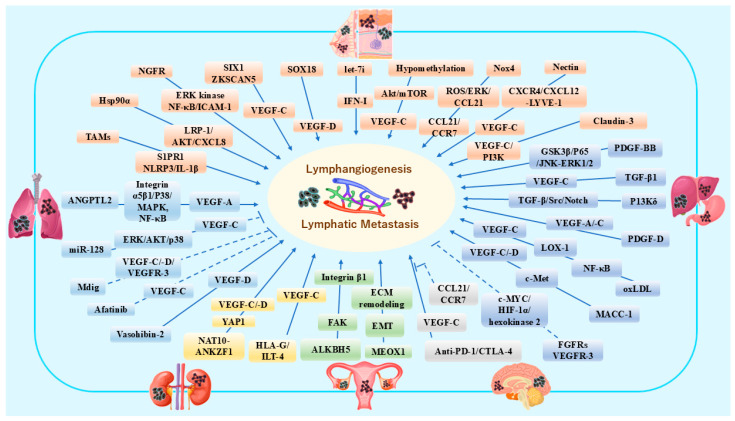
Schematic representation of different growth factors, cytokines, chemokines and other molecules contributing to lymphangiogenesis and lymphatic metastasis in breast cancer and melanoma [86,87,88,89,90,91,92,93,94,95], lung cancer [96,97,98,99,100], gastric cancer and hepatic cancers [101,102,103,104,105,106,107], renal cancer [108,109], ovarian cancer [110,111], and brain malignant tumors [112]. In this figure, six kinds of organs are represented by different colors to reflect the characteristics in organ-specific molecular biology research, e.g., green represents ovarian cancer and related pathways in lymphangiogenesis. The dotted lines indicate inhibitory molecular pathways. This figure is created by Microsoft Office Home & Business 2024 (Windows 11) and Adobe Photoshop Elements & Premiere Elements 2023 (Product Internal Version, 21.0, Windows 11).

A growing body of evidence indicates that abnormal lymphangiogenesis, including different cellular steps, e.g., proliferation, sprouting, migration, and tube formation, might become a target for intervening in tumor development, progression, metastasis, and treatment. Post-developmental lymphangiogenesis in mammals, including rodents and humans, is mainly driven by VEGF-C/VEGFR-3 axis and other signaling pathways in cancer progression and metastasis [70]. Beyond antigen transport, tumor-associated LECs present tumor antigens and express several immune-modulatory signals, displaying different transcriptional and molecular profiles. LECs regulate tumor-specific immune response by affecting the survival, migration, and function of immune cells. In TME, dynamic changes in cellular components, cytokines, and EVs may contribute to pre-metastatic niche formation in different metastatic organs, including LNs. In the following paragraphs, some representative organs with higher cancer incidence and mortality rates [85] will be summarized to emphasize individual pathological and molecular features of lymphangiogenesis in TME (Figure 2).

### 4.1. Breast Cancer and Melanoma

Melanoma and breast cancers are common cancers that preferentially metastasize to regional LNs via lymphatic vessels, especially lymphangiogenesis [113]. Serving as an intrinsic driver for tumor cell crosstalk with TME, the lymphatic system is the main route of breast cancer metastasis, with the assistance of special cytokine expression. Analysis of signal transduction from tumor cells to stromal cells has shown that different cytokines or chemokines are secreted by various types of cells into the microenvironment, which might help to direct their migration route [114]. In human invasive ductal breast carcinoma, sex-determining region Y (SRY)-box transcription factor 18 (SOX18) expression correlates positively with VEGF-D, indicating a possible role of SOX18 in the lymphangiogenesis process [86]. In human invasive breast cancer, TAMs promote tumor lymphangiogenesis via sphingosine-1-phosphate (S1P) receptor 1 and NLR family pyrin domain containing 3 (NLRP3)/IL-1β axis, contributing to LN invasion and metastatic spread [87]. In breast cancer models, SIX1 expression promotes peritumoral and intratumoral lymphangiogenesis, lymphatic invasion, and distant metastasis via upregulation of VEGF-C [88]. Galectin 8 expressed by LECs, promotes the activation of pro-migratory integrin-β1 in breast tumors, facilitating the attachment of TAMs to LECs. TAMs induce lymphangiogenesis and ECM remodeling, consequently increasing tumor lymphatic invasion. Anti-integrin β1, podoplanin knockout, or galectin-8 inhibition impairs TAM adhesion to LECs, and consequently inhibits lymphangiogenesis and lymphatic metastasis [115]. Nectin-4 has been indicated to promote lymphangiogenesis and lymphatic metastasis in breast cancer by regulating the CXCR4/CXCL12-LYVE-1 axis. Nectin-4 induces chemotactic interactions between CXCR4-expressing cancer cells and CXCL12-expressing LECs, which stimulates VEGF-C and LYVE-1 expression to promote LEC proliferation and migration, ultimately promoting lymphangiogenesis [89]. Heat shock protein 90α (Hsp90α) regulates LEC migration and tube formation to promote lymphangiogenesis via LRP1/AKT/CXCL8 signaling pathway [90]. Additionally, lymphangiogenesis induced by platinum chemotherapy increases lymphatic metastasis in breast cancer, which can be prevented by adjuvant anti-VEGFR-3 therapy [116].

The transcription factor zinc finger with KRAB and SCAN domains 5 (ZKSCAN5) activates VEGF-C expression by interacting with suppressor ensemble domain 7 (SETD7) to promote lymphangiogenesis, tumor growth, and metastasis of breast cancer [117]. In 4T1 tumor-bearing mice, NADPH oxidase 4 (Nox4) increases tumor lymphangiogenesis via reactive oxygen species (ROS)/extracellular signal-regulated kinase (ERK)/chemokine CC-ligand 21 (CCL21) pathway and attracts CC chemokine receptor 7 (CCR7)-positive breast cancer cells to entry lymphatic vessels and spread to distant organs [91]. VCAM-1 and its receptor integrin α4 are upregulated by tumor-associated LECs. Disruption of lymphatic junctions and increased permeability via tumor-induced lymphatic VCAM-1 expression may represent a new target to block lymphatic invasion and metastasis [118].

In melanoma and breast cancer mouse models, local ablation of lymphatic vessels results in peritumoral edema, with increased TAMs and other immunosuppressive cells, and high expression of inflammatory cytokines, e.g., TNF-α, IFN-γ, and IL1-β. Tumors grown in lymphatic ablated mice exhibit decreased intratumoral accumulation of cytotoxic T cells and increased tumor PD-L1 expression, which suppresses tumor-specific immune responses and causes rapid tumor growth [119]. Melanoma cells can switch to alternative states under specific microenvironmental stimuli, e.g., TGF-β and/or TNF-α. Melanoma, characterized by increased cellular plasticity, is involved in reversible transcriptional changes emerging from the underlying epigenome [120], which may affect the response to anti-immune checkpoint therapy. In a syngeneic melanoma mouse model, the expression of MHC-I and PD-L1 facilitates LN metastasis by promoting evasion of natural killer cells and T cell suppression. LN metastases resist T cell-mediated cytotoxicity, induce antigen-specific Tregs, and generate tumor-specific immune tolerance that further leads to distant tumor colonization [121]. In this way, LECs may be involved in creating an immunosuppressive environment.

The tumor-draining LNs are considered as survival niches that support T cell priming against lymphatic transported tumor antigen. Thus, the draining LNs may represent a unique potential tumor immunity reservoir for which strategies may be developed to improve immunotherapy effects of immune checkpoint blockade in triple-negative breast cancer [122]. Melanoma-derived EVs transfer tumor antigens to draining LNs mediated by VCAM-1 signaling, and interact with LN-resident LECs and medullary sinus macrophages, contributing to pathological LN remodeling, pre-metastatic niche formation, and immune inhibition [123]. In murine models, melanoma-derived small EVs are enriched in the nerve growth factor receptor (NGFR), which enhances lymphangiogenesis and tumor cell adhesion by inducing ERK kinase, nuclear factor-κB (NF-κB) activation, and intracellular adhesion molecule (ICAM)-1 expression in LECs, and thus reinforcing LN pre-metastatic niche formation and metastasis [92]. High CD147 expression is correlated with the number of lymphatic vessels in human melanoma LNs, and paracrine CD147 is able to upregulate lymphangiogenesis through expression of Prox-1 transcription factor [124]. In murine and human melanoma, apelin promotes lymphangiogenesis, tumor growth, and pulmonary metastases [125]. Rapamycin suppresses lymphangiogenesis in melanoma by blocking mTOR signaling, subsequently downregulating the expression of VEGF-C/VEGFR-3 [126]. Claudin-3 deficiency upregulates tumor lymphangiogenesis by regulating VEGF-C and PI3K signaling pathways, and increases melanoma cell metastasis into the sentinel LNs [93].

Recently, long non-coding RNAs (lncRNAs) have been widely explored in tumorigenesis and progression. A study has identified that lncRNA is highly upregulated in metastatic triple-negative breast cancer, in which hypomethylation promotes tumor cell proliferation, LN metastasis, and lymphangiogenesis by activating forkhead box k1 (FOXK1)-mediated Akt/mTOR and VEGF-C signaling [94]. E26 transformation-specific (ETS) transcription factor ELK3 expressed in LECs promotes breast cancer progression and metastasis through exosomal microRNAs [127]. Melanoma-derived melanosomes facilitate the transfer of mature microRNA lethal-7i (let-7i) to dermal LECs in the early stages of melanoma, which mediate pro-lymphangiogenic phenotypic changes by induction of type I IFN signaling within LECs. Blocking lymphangiogenesis by inhibiting either melanosome release or IFN-I signaling will prevent melanoma progression to the deadly metastatic stage [95].

### 4.2. Lung Cancer

Lymphangiogenesis is considered to be a key initial step in LN metastasis of lung cancer. Tumor lymphangiogenesis is considered to be a novel prognostic indicator for the risk of LN metastasis in non-small cell lung cancer (NSCLC) [128]. Increased M2 subtype of TAMs is positively correlated with VEGF-A/-C expression in human NSCLC, which contributes to tumor lymphangiogenesis [129]. In lung squamous cell carcinoma, vasohibin-2 promotes VEGF-D-induced lymphangiogenesis, and tumor proliferation and invasion [96]. A highly metastatic model of human lung cancer shows increased expression of IL-1 in tumor stromal cells, and increased expression of VEGF-A/-C in M2 TAMs, which provides a highly metastatic TME favorable for lymphangiogenesis and LN metastasis [130].

In NSCLC, microRNA-128, a tumor suppressor, is involved in cancer differentiation and LN metastasis, inhibition of lymphangiogenesis through directly targeting VEGF-C, and blockage of extracellular signal-regulated kinase (ERK), phosphatidylinositol 3-kinase (AKT), and p38 signaling [97]. The adipokine angiopoietin-like protein 2 (ANGPTL2) promotes tumor growth and VEGF-A-dependent lymphangiogenesis via integrin α5β1, p38 MAP kinase (MAPK), and NF-κB signaling in human lung cancer [98]. Integrin-α6 overexpression promotes lymphangiogenesis and then induces lymphatic metastasis via activating NF-κB signaling in lung adenocarcinoma [131]. Afatinib, a tyrosine kinase inhibitor (TKI) of the epidermal growth factor receptor (EGFR) family, prevents tumor growth and lymphangiogenesis via inhibiting VEGF-C secretion in the xenograft mouse model of NSCLC [99]. The mineral dust-induced gene (mdig), an oxygen-sensitive protein, promotes tumor growth but inhibits lymphangiogenesis by blocking expression of VEGF-C/VEGF-D/VEGFR-3 via hypoxia-inducible factor-1α (HIF-1α) signaling in lung adenocarcinoma [100]. TGF-β signaling, acting as a tumor malignant factor in LECs, maintains lymphatic structure and homeostasis, in addition to promoting tumor metastasis. LEC-specific deletion of the TGF-β receptor type II (TGF-β RII) gene results in lymphatic dilation and impaired lymphatic drainage in a mouse model of Lewis lung carcinoma [132], indicating that suppression of TGF-β signaling in LECs might be effective in inhibiting cancer metastasis.

In lung of living body, although it is extremely difficult to directly observe dynamics of lymphatic valves, lymphatic flow, and leukocyte trafficking in intact lymphatic vessels, recently, intravital imaging of pulmonary lymphatic vessels in mice has been developed to reveal migratory DCs following inflammation, and to successfully measure effects of pharmacologic and genetic interventions targeting chemokine signaling, especially to capture lymphatic metastasis and immune surveillance in the lung cancer [133].

### 4.3. Gastric Cancer and Hepatic Cancer

Gastric cancer is one of the most common malignancies worldwide, and increased lymphangiogenesis is thought to be an important step in LN metastasis. In human gastric cancer, the metastasis-associated in colon cancer-1 (MACC1) upregulates VEGF-C/-D via c-mesenchymal–epithelial transition factor (c-Met) signaling to promote lymphangiogenesis, actively contributing to tumor growth and spread [101]. Loss of microRNA-7 promotes p65-mediated aberrant NF-κB activation, facilitating distant metastasis. Delivery of microRNA-7 displays anti-metastatic properties by suppressing VEGF-C-driven lymphangiogenesis and inflammatory infiltration [134]. In gastric cancer models, VEGF-C induced by TGF-β1 signaling enhances tumor-induced lymphangiogenesis, and TGF-β1 receptor inhibitor suppresses tumor progression and lymphangiogenesis [102]. In TME of gastric cancer, overexpression of cysteine-rich intestinal protein-1 (CRIP1) induces lymphangiogenesis and lymphatic permeability, and then facilitates lymphatic metastasis by upregulating expression of VEGF-C and C-C motif chemokine ligand 5 (CCL5) signaling [135]. Sterol O-acyltransferase 1 (SOAT1), a rate-limiting enzyme, can upregulate metabolic genes, the sterol regulatory element binding transcription factor 1 (SREBP1) and SREBP2, inducing lymphangiogenesis via promoting VEGF-C expression. Knockdown or inhibition of SOAT1 suppresses cholesterol ester synthesis, cancer proliferation, LN metastasis, and lymphangiogenesis in gastric cancer [136]. The plasma oxidized low-density lipoprotein (oxLDL) promotes activation of NF-κB signaling via mediating lectin-like oxLDL-1 (LOX-1), and subsequently enhances lymphangiogenesis and lymphatic metastasis by upregulating VEGF-C expression and secretion in gastric cancer cells [103]. Hsa_circ_0000437 promotes pathogenesis of gastric cancer, lymphangiogenesis, and LN metastasis [137]. CD45-erythroid progenitor cells are positively associated with lymphangiogenesis and promote LN metastasis in gastric cancer by inducing a hybrid epithelial/mesenchymal state and LEC migration via secretion of S100A8/A9 [138].

The liver is the largest lymph-producing organ in the body, contributing to tumor dissemination and anti-tumor immunity by regulating lymphangiogenesis. In a mouse model of hepatocellular carcinoma (HCC), overexpression of VEGF-D promotes tumor growth and LN metastasis via lymphangiogenesis [139]. Thrombospondin 1 and 2 (TSP-1, TSP-2), along with pigment epithelium-derived factor (PEDF), promote tumor-associated lymphangiogenesis, and blocking these targets obviously reduces tumor growth and LN infiltration in intrahepatic CCA [140]. In a CCA model, intrinsic cancer cell phosphoinositide 3-kinase δ (PI3Kδ) regulates fibrosis and lymphangiogenesis, and PI3Kδ-mediated cell morphogenesis and ECM remodeling are dependent on TGF-β/Src/Notch signaling [104]. Platelet-derived growth factor-D (PDGF-D) released by CCA cells recruits and activates CAFs that secrete VEGF-A/-C to drive lymphangiogenesis and lymphatic invasion [105]. In intrahepatic CCA, inhibition of dual fibroblast growth factor receptors (FGFRs) and VEGFR-3 synergistically restrains lymphangiogenesis and improves antitumor immunocompetence through suppression of c-myelocytomatosis oncogene (c-MYC)-dependent and HIF-1α-mediated hexokinase 2 expression, respectively [106]. CAFs-derived PDGF-BB activates the glycogen synthase kinase 3β (GSK3β)/P65 pathway to promote trans-endothelial migration of tumor cells, and stimulates downstream c-Jun N-terminal kinase (JNK) and ERK1/2 pathways to induce intratumoral lymphangiogenesis [107], which suggests that targeting PDGF-BB/PDGF receptor β can inhibit tumor growth and lymphatic metastasis in CCA.

### 4.4. Renal Cancer

Lymphangiogenesis is potentially involved in the progression and metastasis of clear cell renal cell carcinoma (ccRCC), representing a promising therapeutic target. Sunitinib, an anti-angiogenic drug, stimulates expression of VEGF-C by tumor cells and promotes lymphangiogenesis and LN metastasis in experimental and human ccRCC [141], suggesting that a therapy for inhibiting tumor blood vessels but inducing lymphatic growth may eventually result in treatment failure. Androgen receptor plays a dual role in determining metastatic patterns of ccRCC, through differential regulation of VEGF-A or VEGF-C expression via miR-185-5p, which affects angiogenesis or lymphangiogenesis, and ultimately increases hematogenous but decreases lymphatic metastases at different sites [142]. Interferon-induced transmembrane protein 2 (IFITM2), a novel p53-independent pro-apoptotic gene, may promote tumor progression via inducing cytokine release. In ccRCC, upregulated IFITM2 promotes lymphatic metastasis and lymphangiogenic activity via VEGF-C signaling [143]. *N*-acetyltransferase 10 (NAT10)-ankyrin repeat and zinc finger peptidyl tRNA hydrolase 1 (ANKZF1) axis promotes tumor progression and lymphangiogenesis of ccRCC by enhancing the nuclear import of Yes1-associated transcriptional regulator (YAP1), and upregulating expression of VEGF-C/-D [108]. In human samples of ccRCC, high expression of human leukocyte antigen G (HLA-G) and its receptor, immunoglobulin-like transcript-4 (ILT-4), is consistent with the formation of an immune-tolerant microenvironment. The immune-checkpoint HLA-G/ILT4 interaction increases VEGF-C expression in ccRCC [109], suggesting that it can provide an effective signal transmission to form an immunosuppressive TME, and induce tumor-associated lymphangiogenesis and subsequent lymphatic metastasis.

Lymphangiogenesis influences both tumor progression and chronic transplant rejection, where lymphatic progenitor cells may derive from stromal macrophages and then incorporate into growing LECs [144]. Macrophages are greatly involved in lymphangiogenesis in renal disease models, and activation of VEGF-C/VEGFR-3 signaling pathway can promote phenotypic changes through downregulation of autophagy. This pathological lymphangiogenesis may originate from classically activated macrophage (M1) polarization and transdifferentiation into LECs [71,145].

### 4.5. Ovarian Cancer

Excess lymphangiogenesis facilitates aggressive metastatic spread and ascites development in ovarian cancer, the second most common gynecological cancer [146]. In ovarian carcinoma xenografts, ovariectomy increases gonadotropin levels and induces VEGF-C promoter activation and tumor lymphangiogenesis, whereas VEGF-C expression is mediated by lens epithelium-derived growth factor (LEDGF) [147]. A paracrine Hedgehog signaling in CAFs is indicated to accelerate lymphangiogenesis in a murine xenograft model. Ovarian cancer cell-derived Sonic Hedgehog (SHH) induces VEGF-C expression in CAFs that constitute a supportive niche for tumorigenesis and lymphangiogenesis [148]. ScRNA-seq-based TME analysis has indicated that specific cell subtypes influence survival and determine molecular subtype classification, in which TGF-β-driven CAFs and LECs are correlating with poor outcome in high-grade serous tubo-ovarian cancer patients [149]. In addition to microRNAs, RNA modification is involved in post-transcriptional regulation of lymphangiogenesis. AlkB homolog 5 (ALKBH5) overexpression activates focal adhesion kinase (FAK) signaling through N6-methyladenosine demethylation in integrin β1 mRNA, and enhances tumor-associated lymphangiogenesis and LN metastasis in ovarian cancer [110]. Recently, overexpression of mesenchyme homeobox 1 (MEOX1), a transcriptional factor controlling somite development, has been found to be involved in the occurrence and progression of ovarian cancer by regulating proliferation and EMT of cancer cells, lymphangiogenesis, and ECM remodeling [111].

In mouse cancer models, administration with anlotinib, a tyrosine kinase inhibitor with anti-lymphangiogenesis activity, and SAR131675, a selective VEGFR-3 inhibitor, effectively decreases LVD and interstitial fluid pressure in tumor tissues, which may improve anti-tumor efficacy by increasing intratumoral accumulation of nanoparticle, macromolecule, and small molecule therapeutic drugs [81]. The anti-lymphangiogenesis strategy may reduce lymphatic metastasis and elicit anti-tumor immune responses. Rapid development in lymphangiogenesis studies has become an important driving force for accelerating the discovery of therapeutic agents targeting cancer metastasis [150].

### 4.6. Brain Malignant Tumors

Functional lymphatic vessels lining the dural sinuses express all of the molecular hallmarks of LECs, which carry fluid and immune cells from CSF, and drain into the deep cervical LNs [151]. VEGF-C-dependent meningeal lymphangiogenesis surrounding the central nervous system (CNS), postnatally characterized by lymphatic sprout extension and cluster fusion, is involved in CSF clearance and some neuropathological processes [42,152]. Meningeal lymphangiogenesis occurs in the alymphatic zone lateral to the sagittal sinus after photothrombosis-induced stroke in Vegfr3wt/wt mice. The study has indicated that ischemic injury can induce meningeal lymphatic growth through VEGFR-3 signaling, and absence of the lymphatic vessels can impact post-stroke outcomes [153]. Meningeal lymphangiogenesis promotes the clearance of amyloid β in a mouse model of Alzheimer’s disease via immunotherapy with anti-amyloid β antibodies [154]. Intracranial VEGF-C administration induces dural lymphangiogenesis in mice, leading to improved clearance of CSF macromolecules into cervical LNs. However, sustained meningeal lymphatic atrophy or expansion during this process does not affect amyloid-β deposition in the brain [155], suggesting that other compensatory pathways may promote CSF clearance.

Meningeal lymphatic vessels are essential in generating an efficient immune response against brain tumors. In a striatal tumor model, VEGF-C overexpression induces meningeal lymphangiogenesis, displaying a synergy effect with anti-PD-1/cytotoxic T-lymphocyte-associated protein 4 (CTLA-4) therapy, which can be abolished by CCL21/CCR7 blockade [112]. The results indicate that VEGF-C potentiates checkpoint therapy via CCL21/CCR7 signaling. Moreover, VEGF-C/VEGFR-3 signaling inhibition by VEGFR-3 monoclonal antibody, a soluble VEGF-C/-D trap, or deletion of Vegfr3 gene in adult mice leads to notable regression and functional impairment of dural lymphatic vessels, but without effect on CNS autoimmunity development [156]. In a mouse model of glioblastoma multiforme, ectopic VEGF-C expression promotes CD8^+^ T cell infiltration, and antigen drainage into tumor tissues and cervical LNs. VEGF-C administration in combination with anti-PD-1 immunotherapy suppresses tumor growth and prolongs survival [80]. The meningeal lymphangiogenesis is beneficial in increasing the efficacy of checkpoint inhibitor treatment for glioblastoma.

In tumor tissues, various factors affect enhanced permeability and retention (EPR) effect, e.g., tumor perfusion, lymphatic function, interstitial penetration, vascular permeability, nanoparticle retention [157]. Lymphangiogenesis involves pre-existing LEC sprouting, migration, proliferation, and tubular formation, mainly depending on VEGF-C and VEGFR-3 signaling. Currently, molecular and genetic techniques have been utilized to explore potential therapeutic targets for modulating lymphangiogenesis in cancers. Therefore, this article has addressed recent research advances of the process of lymphangiogenesis in TME, with emphasizing molecular involvement and biofunctional characteristics of organ-associated LECs (Table 1).

Furthermore, the lymphangiogenesis-chip platform permits the interrogation of various lymphatic biological functions and lymphatic immunomodulation for cancer therapy [158]. The development of the lymphangiogenesis-chip may provide an important opportunity for studying heterotypic and plastic characteristics, and interactions of LECs with other cell components, e.g., cancer cells, immune cells, and other stromal cells, in which CAF-derived EVs, as biomarkers and drug carriers, still remain an attractive target for modulating tumor-associated lymphangiogenesis and cancer therapy. Finally, organ-associated lymphangiogenesis is regulated by a wide variety of signaling pathways in TME. The efficacy of regulatory non-coding RNAs (miRNAs, circRNAs), various neutralizing antibodies, and tyrosine kinase inhibitors, which serve as critical modulators of lymphangiogenesis, has been evaluated so far in animal models and preclinical trials. Therefore, the intervention of organ-specific lymphangiogenesis, especially the VEGF-C/VEGFR-3 signaling, may be promising for molecular target and gene therapy.

## 5. Conclusions and Perspectives

**Figure 3:**
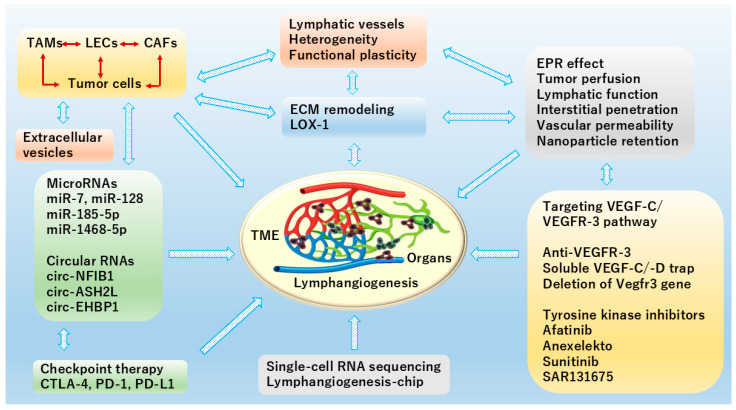
Schematic diagram of organ-specific TME contributing to lymphangiogenesis illustrates recent popular topics in CAFs and ECM remodeling as well as extracellular vesicles, application of microRNAs (miRNA) and circular RNAs (circRNAs) in cancer, regulation of VEGF-C/VEGFR-3 signaling, and EPR effect. In TME, miRNAs and circRNAs play crucial and complex roles by acting as key regulators of cellular communication, immune modulation, and lymphatic metastasis. Mostly, they inhibit tumor metastasis via reducing VEGF-C secretion or directly targeting VEGF-C to suppress lymphangiogenesis. Meanwhile, ScRNA-seq for profiling gene expression differences on a complex cellular level, and lymphangiogenesis-chip for serving as a disease and drug discovery model are also included here. This figure is created by Microsoft Office Home & Business 2024 (Windows 11) and Adobe Photoshop Elements & Premiere Elements 2023 (Product Internal Version, 21.0, Windows 11).

Increased evidence has shown that multiple signaling pathways, especially the VEGF-C/VEGFR-3 axis, affect cancer progression and therapy by controlling lymphangiogenesis in different organs. This review provides an overview of current knowledge regarding the regulation of lymphangiogenesis, emphasizing LEC heterogeneity, lymphatic functional diversity and plasticity, and ECM remodeling in TME. LECs show distinct immunomodulatory gene expression profiles and offer new targets for controlling antigen presentation, immune cell migration, and cancer metastasis. The dual role of lymphatic vessels in cancer metastasis and immunotherapy will further deepen understanding of organ-associated lymphangiogenesis. In this field, ScRNA-seq technology has accelerated novel discoveries in lymphatic biology, and the lymphangiogenesis-chip may serve as a disease and drug discovery model for preclinical and translational studies in lymphatic-associated diseases. A better understanding of a variety of traits and behaviors of LECs, immune cells, and stromal cells like CAFs may lead to new therapeutic interventions in cancers (Figure 3).

## Figures and Tables

**Table 1:** Organ-associated LECs are linked with biofunctional characteristics in the process of lymphangiogenesis and molecular involvement in TME.

Cancers	Animal/ Human	Molecules	Biofunctional Characteristics	References
Breast cancer	Mouse Human	Galectin 8 Integrin β1 Podoplanin	Peri-lymphatic TAM localization is mediated by galectin 8 expression in LECs Podoplanin-expressing macrophages promote lymphangiogenesis and tumor invasion, and their migration and adhesion depend on galectin 8-mediated integrin β1 activation	[115]
Breast cancer	Mouse	VCAM-1	VCAM-1 and integrin α4 are upregulated by LECs VCAM-1 regulates lymphatic invasion and permeability through junction remodeling	[118]
Breast cancer	Mouse	ELK3	ELK3 expressed in LECs promotes cancer progression and metastasis through exosomal miRNAs	[127]
Breast cancer	Mouse	VEGFR-3 inhibitor (SAR131675) Anlotinib	Anti-lymphangiogenesis therapy mediated by anlotinib and SAR131675 and improves anti-tumor efficacy through increasing intratumoral accumulation of nanoparticles, macromolecules, and small molecular drugs	[81]
Breast cancer Melanomas	Mouse	TNF-α, IFN-γ IL-1β TGF-β1, IL10 PD-L1	Lymphatic ablation increases inflammatory cell accumulation and tumor growth Excess inflammatory cytokines (TNF-α, IFN-γ, IL-1β), and immunosuppressive molecule PD-L1 and cytokines (TGF-β, IL10) are noted in peritumoral edematous fluid	[119]
Melanoma	Mouse Human	NGFR	Melanoma sEV-secreted NGFR promotes tumor cell adhesion and lymphangiogenesis sEVs taken up by LECs and macrophages, reinforce LN pre-metastatic niche formation and metastasis	[92]
Melanomas	Mouse	VEGF-C	VEGF-C overexpression in irradiated tumor cell vaccines promotes T cell priming in the injection site and draining LNs Lymphangiogenic vaccines elicit a strong melanoma-specific T cell immunity and provide effective tumor control and long-term immunological memory	[66]
Melanomas	Mouse	VCAM-1	Uptake of B16F10-derived EVs by LN LECs is mediated by VCAM-1 EVs induce lymphatic remodeling and tumor antigen cross-presentation by LECs in draining LNs	[123]
NSCLC	Human	VEGF-A VEGF-C	High expression of M2 ratio is an indicator of poor prognosis, positively correlated with VEGF-A and VEGF-C Increased M2 TAMs contribute to tumor lymphangiogenesis and progression by promoting VEGF-C expression	[129]
Lung adenocarcinoma	Mouse Human	Integrin α6	Integrin α6 overexpression promotes lymphangiogenesis and lymphatic metastasis via activating NF-κB signaling	[131]
Gastric cancer	Mouse Human	MicroRNA-7 NF-κB RelA/p65	miR-7 inhibits gastric metastasis by suppressing lymphangiogenesis via reducing VEGF-C secretion Loss of miR-7 in gastric cancer promotes RelA/p65-mediated aberrant NF-κB activation, facilitating tumor metastasis	[134]
Gastric cancer	Human	SOAT1 SREBP1/2 VEGF-C	The expression of SREBP1 and SREBP2 regulated by SOAT1 induces lymphangiogenesis via increasing VEGF-C expression Knockdown or inhibition of SOAT1 suppresses cancer proliferation and LN metastasis	[136]
Gastric cancer	Mouse Human	S100A8/A9	CD45-erythroid progenitor cells increase lymphangiogenesis and promote LN metastasis through S100A8/A9 heterodimer-induced hybrid epithelial/mesenchymal state in LECs	[138]
Gastric cancer	Mouse Human	CRIP1 CCL5 VEGF-C	CRIP1 promotes VEGF-C secretion, lymphangiogenesis and lymphatic metastasis CRIP1 increases LVD and lymphatic permeability via CCL5-mediated recruitment of TAMs and TNF-α secretion	[135]
CCA	Rat Mouse Human	PDGF-D VEGF-A/-C	PDGF-D stimulates fibroblasts to secret VEGF-A/-C PDGF-D enables liver myofibroblasts to promote tumor lymphangiogenesis	[105]
CCA	Mouse Human	PDGF-BB/ PDGFR-β	CAFs promote lymphangiogenesis and lymphatic metastasis via PDGF-BB/PDGFR-β-mediated signaling	[107]
Intrahepatic CCA	Mouse Human	THBS1 THBS2 PEDF	Overexpression of THBS1, THBS2 and PEDF promotes tumor-associated lymphangiogenesis Blocking THBS1, THBS2, and PEDF reduces tumor growth and LN infiltration	[140]
Intrahepatic CCA	Mouse Human	PI3Kδ TGF-β	PI3Kδ promotes tumor growth, aggressiveness and ECM remodeling, as well as lymphangiogenesis via TGF-β/Src/Notch signaling	[104]
Intrahepatic CCA	Mouse Human	FGFRs VEGFs	FGFR-1 and VEGFR-3 expression induce lymphangiogenesis and lymphatic metastasis Dual FGFR and VEGFR inhibition inhibits lymphangiogenesis by suppression of c-MYC-dependent and HIF-1α-mediated hexokinase-2 expression, and improves antitumor immunity by downregulating PD-L1 expression in LECs	[106]
ccRCC	Mouse Human	miR-185-5p VEGF-C	Androgen receptor decreases lymphangiogenesis and lymphatic metastasis through suppressing VEGF-C expression by upregulating miR-185-5p	[142]
ccRCC	Human	IFITM2 VEGF-C	IFITM2-VEGF-C signaling contributes to enhanced lymphangiogenesis and lymphatic metastasis	[143]
ccRCC	Mouse Human	ANKZF1 NAT10 VEGF-C/-D	NAT10-ANKZF1 axis promotes tumor progression and lymphangiogenesis, by enhancing YAP1 nuclear import and VEGF-C/-D expression	[108]
Ovarian cancer	Mouse Human	SHH VEGF-C	Paracrine signaling of tumor-initiated SHH promotes lymphangiogenesis via CAF-derived VEGF-C	[148]
Ovarian cancer	Mouse Human	CircASH2L miR-665 VEGF-A	CircASH2L promotes ovarian cancer tumorigenesis and lymphangiogenesis by regulating the miR-665/VEGF-A axis	[32]
Ovarian cancer	Mouse Human	ALKBH5 FAK	ALKBH5 activates FAK signaling, and enhances lymphangiogenesis and LN metastasis	[110]
Striatal tumors (Intracranial gliomas)	Mouse	anti-PD-1/ CTLA-4 CCL21/CCR7 VEGF-C	Intracranial tumors induce remodeling of dorsal meningeal lymphatic vessels VEGF-C-induced lymphangiogenesis is required for dendritic cell trafficking to deep cervical LNs Immunotherapy enhancement by VEGF-C is dependent on CCL21/CCR7 signaling Lymphatic ablation impairs anti-PD-1/CTLA-4 efficacy against tumors	[112]
Glioblastoma (Brain tumor)	Mouse Human	VEGF-C anti-PD-1	VEGF-C/anti-PD-1 immunotherapy suppresses tumor growth and prolongs survival Therapeutic delivery of VEGF-C potentiates checkpoint inhibitor therapy by increasing T cell priming Glioblastoma microenvironment is devoid of lymphangiogenic signals	[80]

## Data Availability

No new data were created or analyzed in this study.

## Abbreviations

AKT, phosphatidylinositol 3-kinase; ALKBH5, AlkB homolog 5; ANGPTL2, adipokine angiopoietin-like protein 2; ANKZF1, Zinc finger peptidyl tRNA hydrolase 1; Anti-β2-GPI antibody, anti-β2 glycoprotein I antibody; ARG-1, arginase-1; ATG5, autophagy-related gene 5; AXL, anexelekto; BEC, blood endothelial cell; CAFs, cancer-associated fibroblasts; CCA, cholangiocarcinoma; CCBE1, collagen and calcium-binding EGF domain-1; CCL5, C-C motif chemokine ligand 5; CCL21, chemokine CC-ligand 21; CCR7, CC chemokine receptor 7; ccRCC, clear cell renal cell carcinoma; circRNAs, circular RNAs; circASH2L, circular RNA absent-small-homeotic-2-like protein; CLEC11A, c-type lectin domain containing 11A; c-Met, c-mesenchymal–epithelial transition factor; c-MYC, c-myelocytomatosis oncogene; CNS, central nervous system; CRIP1, cysteine-rich intestinal protein-1; CTLA-4, cytotoxic T-lymphocyte-associated protein 4; EAF-2, eleven-nineteen lysine-rich leukemia-associated factor-2; ECs, endothelial cells; ECM, extracellular matrix; EGFR, epidermal growth factor receptor; EMT, epithelial–mesenchymal transition; EndMT, endothelial-to-mesenchymal transition; EPR, enhanced permeability and retention; ERK, extracellular signal-regulated kinase; ETS, E26 transformation-specific; EVs, extracellular vesicles; DLL4, delta-like protein 4; FAK, focal adhesion kinase; FGFRs, fibroblast growth factor receptors; Foxc2, forkhead box protein C2; FOXK1, forkhead box k1; FSP1, fibroblast-specific protein-1; GSK3β, glycogen synthase kinase 3β; HCC, hepatocellular carcinoma; HIF-1α, hypoxia-inducible factor-1α; HLA-G, human leukocyte antigen G; Hsp90α, heat shock protein 90α; ICAM, intracellular adhesion molecule; IDO, indoleamine 2,3-dioxygenase; IFITM2, interferon-induced transmembrane protein 2; IFN-γ, interferon-γ; IL-35, interleukin-35; ILT-4, immunoglobulin-like transcript-4; iNOS, inducible nitric oxide synthase; IRF2, interferon regulatory factor 2; JAM-2, junctional adhesion molecule-2; LECs, lymphatic endothelial cells; LEDGF, lens epithelium-derived growth factor; let-7i, mature microRNA lethal-7i; LGR5, leucine-rich repeat-containing G-protein-coupled receptor 5; lncRNAs, long non-coding RNAs; LNs, lymph nodes; LOX-1, lectin-like oxLDL-1; LRP8, low-density lipoprotein receptor-related protein 8; LVD, lymphatic vessel density; MACC1, metastasis-associated in colon cancer-1; MAPK, MAP kinase; Mdig, mineral dust-induced gene; MEndT, mesenchymal-to-endothelial transition; MEOX1, mesenchyme homeobox 1; MHC-II, major histocompatibility complex class II; mregDCs, mature dendritic cells enriched in immunoregulatory molecules; NAT10, *N*-acetyltransferase 10; NGFR, nerve growth factor receptor; NOS2, nitric oxide synthase 2; Nox4, NADPH oxidase 4; NSCLC, non-small cell lung cancer; oxLDL, oxidized low-density lipoprotein; PD-1, programmed cell death 1; PDGF, platelet-derived growth factor; PD-L1, programmed cell death-ligand 1; PEDF, pigment epithelium-derived factor; PI3Kδ, phosphoinositide 3-kinase δ; Prox-1, prospero homeobox 1; ROS, reactive oxygen species; S1P, sphingosine-1-phosphate; ScRNA-seq, single-cell RNA sequencing; SETD7, suppressor ensemble domain 7; SHH, sonic Hedgehog; SOAT1, sterol O-acyltransferase 1; SOX18, SRY-box transcription factor 18; SREBP, sterol regulatory element binding transcription factor; SRY, sex-determining region Y; TAMs, tumor-associated macrophages; TGF-β1, transforming growth factor-β1; TGFβR, transforming growth factor-β receptor; TKI, tyrosine kinase inhibitor; TLS, tertiary lymphoid structure; TME, tumor microenvironment; TNF-α, tumor necrosis factor-α; Tregs, regulatory T cells; TSP, thrombospondin; VCAM-1, vascular cell adhesion molecule-1; VEGF-C, vascular endothelial growth factor-C; YAP1, Yes1-associated transcriptional regulator; ZKSCAN5, Zinc finger with KRAB and SCAN domains 5.
